# Supplementary material for: Identification of photoperiod-induced specific miRNAs in the adrenal glands of Sunite sheep (Ovis aries)
Source: Front Vet Sci. 2022 Jul 22;9:888207. doi: 10.3389/fvets.2022.888207 (PMC9354845; doi:10.3389/fvets.2022.888207)
Supplement: Supplementary file 1 [file Data_Sheet_1.ZIP › Supplementary file/miRDeep2 file.zip!/miRDeep2 file/Know_Structure/SP42A2/oar-mir-487a.pdf]

miRBase precursor : oar-mir-487a
Total read count : 136
oar-miR-487a-5p read count : 29
oar-miR-487a-3p read count : 107
remaining reads : 0

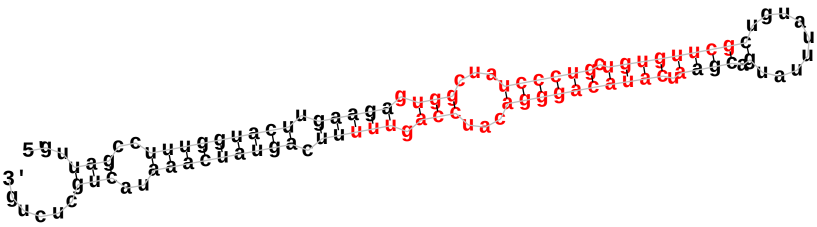

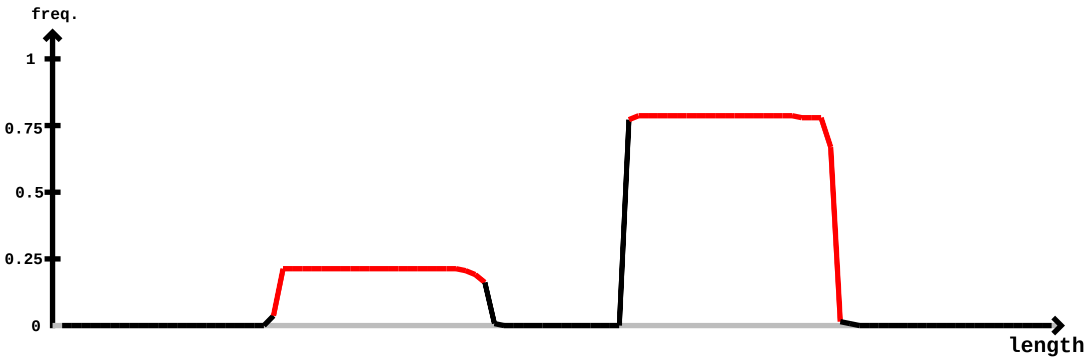


```
                                                               oar-miR-487a-3p
                          oar-miR-487a-5p
5'- guuagccuuugguacuugaagaguggcuaucccugcuguguucgcuguauuuaugacgaaucauacagggacauccaguuuuucaguaucaaauacugcucug   -3'    exp
    ..(((..(((((((((.(((((.(((...((((((.(((((((((.........).)))).))))))))))...))).))))).)))))))))..))).....   reads      mm        sample
    .....................aguggcuaucccugcuguguuc............................................................   4          0         seq
    .....................aguggcuaucccugcuguguucg...........................................................   1          0         seq
    ......................guggcuaucccugcugugu..............................................................   1          0         seq
    ......................guggcuaucccugcuguguu.............................................................   2          0         seq
    ......................guggcuaucccugcuguguucg...........................................................   20         0         seq
    ......................guggcuaucccugcuguguucgc..........................................................   1          0         seq
    ...........................................................aucauacagggacaucc...........................   1          0         seq
    ...........................................................aucauacagggacauccagu.......................   15         0         seq
    ...........................................................aucauacagggacauccaguu......................   88         0         seq
    ...........................................................aucauacagggacauccaguuu.....................   1          0         seq
    ............................................................aucauacagggacauccaguu......................   1          0         seq
    ............................................................aucauacagggacauccaguuuu....................   1          0         seq
```
